# Strong segregation promotes self-destructive cooperation

**DOI:** 10.1093/ismejo/wraf043

**Published:** 2025-03-16

**Authors:** Lingling Wen, Yang Bai, Yunquan Lan, Yaxin Shen, Xiaoyi She, Peng Dong, Teng Wang, Xiongfei Fu, Shuqiang Huang

**Affiliations:** State Key Laboratory of Quantitative Synthetic Biology, Shenzhen Institute of Synthetic Biology, Shenzhen Institutes of Advanced Technology, Chinese Academy of Sciences, No. 1068 Xueyuan Avenue, Nanshan District, Shenzhen 518055, China; University of Chinese Academy of Sciences, No. 1 Yanqihu East Rd, Huairou District, Beijing, 101408, China; State Key Laboratory of Quantitative Synthetic Biology, Shenzhen Institute of Synthetic Biology, Shenzhen Institutes of Advanced Technology, Chinese Academy of Sciences, No. 1068 Xueyuan Avenue, Nanshan District, Shenzhen 518055, China; Shenzhen Infrastructure for Synthetic Biology, Shenzhen Institute of Synthetic Biology, Shenzhen Institute of Advanced Technology, Chinese Academy of Sciences, No. 1068 Xueyuan Avenue, Nanshan District, Shenzhen 518055, China; State Key Laboratory of Quantitative Synthetic Biology, Shenzhen Institute of Synthetic Biology, Shenzhen Institutes of Advanced Technology, Chinese Academy of Sciences, No. 1068 Xueyuan Avenue, Nanshan District, Shenzhen 518055, China; University of Chinese Academy of Sciences, No. 1 Yanqihu East Rd, Huairou District, Beijing, 101408, China; State Key Laboratory of Quantitative Synthetic Biology, Shenzhen Institute of Synthetic Biology, Shenzhen Institutes of Advanced Technology, Chinese Academy of Sciences, No. 1068 Xueyuan Avenue, Nanshan District, Shenzhen 518055, China; Institute of Biomedical and Health Engineering, Shenzhen Institutes of Advanced Technology, Chinese Academy of Sciences, No. 1068 Xueyuan Avenue, Nanshan District, Shenzhen 518055, China; State Key Laboratory of Quantitative Synthetic Biology, Shenzhen Institute of Synthetic Biology, Shenzhen Institutes of Advanced Technology, Chinese Academy of Sciences, No. 1068 Xueyuan Avenue, Nanshan District, Shenzhen 518055, China; State Key Laboratory of Quantitative Synthetic Biology, Shenzhen Institute of Synthetic Biology, Shenzhen Institutes of Advanced Technology, Chinese Academy of Sciences, No. 1068 Xueyuan Avenue, Nanshan District, Shenzhen 518055, China; University of Chinese Academy of Sciences, No. 1 Yanqihu East Rd, Huairou District, Beijing, 101408, China; State Key Laboratory of Quantitative Synthetic Biology, Shenzhen Institute of Synthetic Biology, Shenzhen Institutes of Advanced Technology, Chinese Academy of Sciences, No. 1068 Xueyuan Avenue, Nanshan District, Shenzhen 518055, China; University of Chinese Academy of Sciences, No. 1 Yanqihu East Rd, Huairou District, Beijing, 101408, China

**Keywords:** self-destructive cooperation, microbial evolution, group selection, synthetic biology, evolutionary biology

## Abstract

Self-destructive cooperators, which sacrifice themselves for others, challenge traditional group selection theory, as costs often exceed individual benefits. We predict self-destructive cooperators can persist in highly segregated environments where populations are primarily divided into homogenous groups originating from one or two founders. In such contexts, the benefits of self-destructive cooperators remain within homogeneous groups of self-destructive cooperators, preserving the sacrifice value and ensuring its maintenance. To validate our hypothesis, we employ a synthetic self-destructive cooperators-cheaters system and develop automated experiments to monitor and operate the subgroups with diverse growth behaviors due to strong segregation. Ultimately, we demonstrate self-destructive cooperators is maintained under strong segregation. High stress further enhances self-destructive cooperators by reducing the benefits received by cheaters in heterogeneous subgroups. This study advances group selection theory and automation in evolutionary research.

## Introduction

Self-destructive cooperation represented a high-level manifestation of altruism, where an individual sacrificed himself to produce public goods that ultimately benefited all other members of society [1–5]. Such noble behavior was not limited to humans with heightened consciousness and was also evident in more primitive organisms [6]. Examples included *Escherichia coli* expelling colicins to protect kin [7], honeybees stinging to defend their hives [8], and the immune system’s sacrificial response to sepsis [9]. Such self-destructive cooperative behavior in organisms posed a profound evolutionary puzzle: Because self-destructive cooperation inherently reduced individual fitness, how could they persist in natural selection, a process driven by the imperatives of survival and reproduction.

Traditional group selection theory had successfully explained the maintenance of mild cooperative behaviors of primitive organisms under structured environments. This was predicated on the notion of “multi-level selection”, that cooperators could enhance the productivity of subgroups with a higher composition of cooperators, yielding a net advantage at the group level despite the intra-group disadvantage compared to non-cooperating counterparts [10–13]. However, such a theory failed to explain the evolution of extreme cooperation such as self-destructive cooperation, given that the self-destructive cooperators (SDCs) completely sacrificed themselves, leaving no offspring in subgroups, creating an extreme disadvantage within the group, where potential benefits at the group level might not compensate the cost [13]. Current theories suggested that self-destructive cooperation was not a directly selected trait but an unintended consequence of another beneficial function [14, 15].

In this study, we expanded group selection theory by incorporating the stochastic effects of random sampling, providing new insights into the evolution of self-destructive cooperation (SDC) in structured environments. Using the “Segregation-Growth-Stress-Pool” (SGSP) processes, we theoretically demonstrated that SDC can persist under strong segregation conditions, where group sizes were as minimal as approximately one or two individuals per subgroup. This scenario mirrored the life cycle of biofilms, where dense bacterial communities develop from a limited number of initial cells and expand until encountering environmental stress [16–26], suggesting SDCs might naturally evolve under such conditions. These results were further validated experimentally using a synthetic SDC-cheater system, which included a self-destructive strain engineered to release public goods [2] and a cheater strain that neither self-destructed nor produced public goods. By employing automated experiments through biofoundry technology, which streamlined and executed all the SGSP procedure automatically (see Materials and Methods), we successfully addressed the challenges posed by the highly diverse growth dynamics of tightly segregated subpopulations [27]. We also found that the lower initial composition of SDC necessitated weaker segregation strength for its maintenance, and higher stress levels favored the persistence and proliferation of self-destructive cooperation. These findings not only extended classic group selection theory but also demonstrated the significant potential of biofoundries in advancing our understanding of complex evolutionary phenomena.

## Materials and methods

### Conditions for self-destructive cooperation evolution

Extreme dilution, caused by random sampling of bacterial cells, could have led to strong segregation and a Poisson distribution of isolated groups with varying compositions [28, 29]. In a Poisson random selection process, where subgroups were formed with an average population size $\lambda$ from a large population containing both SDCs and cheaters with initial SDC ratio ${R}_0$, the frequencies of homogeneous subgroups of SDCs ${f}_{homo}^{SDC}$ and cheaters ${f}_{homo}^{cheater}$ were given by: ${f}_{homo}^{SDC}={\sum}_{k=1}^{\infty }{R}_0^k\cdotp \frac{\lambda^k}{k!}\cdotp{e}^{-\lambda }$, ${f}_{homo}^{cheater}={\sum}_{k=1}^{\infty }{\left(1-{R}_0\right)}^k\cdotp \frac{\lambda^k}{k!}\cdotp{e}^{-\lambda }$, where $k$ was a positive integer representing the number of cells in a subgroup. The frequency of empty subgroups was $\mathrm{given}\ \mathrm{by}:{f}_{empty}={e}^{-\lambda }$. Thus, the frequency of heterogeneous subgroups ${f}_{het}$ was the remainder of the non-empty subgroups: ${f}_{het}=1-{f}_{empty}-{f}_{homo}^{SDC}-{f}_{homo}^{cheater}$.

The final SDC ratio after the SGSP process could be calculated by the yields and frequencies of each type of subgroup. We first defined the yields of different subgroups as follows: ${Y}_{homo}^{SDC}$ and ${Y}_{homo}^{cheater}$ represented the yields of homogeneous SDC and cheater groups, respectively, and ${Y}_{het}^{SDC}$ and ${Y}_{het}^{cheater}$represented the yield of SDCs and cheaters in heterogeneous groups. Using these yields, we derived ${R}^{\prime }$ as the ratio of the total yield of SDCs to the total yield of the entire population: ${R}^{\prime }=\frac{f_{homo}^{SDC}{Y}_{homo}^{SDC}+{f}_{het}{Y}_{het}^{SDC}}{f_{homo}^{SDC}{Y}_{homo}^{SDC}+{f}_{homo}^{cheater}{Y}_{homo}^{cheater}+{f}_{het}{Y}_{het}^{SDC}+{f}_{het}{Y}_{het}^{cheater}}.$

We considered the extreme case of altruism, where the SDCs completely sacrificed themselves in heterogeneous groups (${Y}_{het}^{SDC}=0$) whereas cheaters in homogeneous groups were entirely eliminated by stress (${Y}_{homo}^{cheater}=0$). To exclude the possibility of frequency-dependent selection, we assumed all heterogeneous groups shared the same yield for cheaters, which was also equal to the yield of SDCs in homogeneous groups (${Y}_{het}^{cheater}={Y}_{homo}^{SDC}$). Although these simplifications might not hold universally, they were applicable to populations that have undergone strong segregation. Under these simplifications, the final ratio of SDCs was given by: ${R}^{\prime }=\frac{f_{homo}^{SDC}}{f_{homo}^{SDC}+{f}_{het}}$. The positive change in the SDC ratio after the SGSP process, defined as $\Delta R={R}^{\prime }-{R}_0>0$, determined the condition of SDC maintenance: $\frac{f_{homo}^{SDC}}{f_{het}}\ge \frac{R_0}{1-{R}_0}$.

### Cell strains

SDC, NPD, and cheater strains were derived from the *E. coli* SN0301 strain (*ampD1, ampA1, ampC8, pyrB, recA,* and *rpsL*), which the *ampD1* mutation enabled hyper-induction of P*_ampC_* in response to beta-lactam antibiotics [30]. The SDC strain was transformed with the pBlaM plasmid, pCSaE450C plasmid, and a GFP reporter. The NPD strain was transformed with the pBlaM plasmid, pTS1 [31], and a mCherry reporter. The cheater strain was transformed with pPROLar. A122 (Clonetech), pTS1 [31], and a mCherry reporter. The pBlaM plasmid encoded the *BlaM* gene under the control of the P*_lac/ara-1_* promoter from pPROLar. A122, which had a p15A origin of replication. The pCSaE450C plasmid encoded the *E* gene under the P*_ampC_* promoter through AmpR and was based on pTS1 with a pCDF13 origin of replication. The fluorescent reporters (GFP or mCherry) were carried on plasmids based on pZS31 with a pSC101 origin of replication.

### Culture medium

Unless otherwise noted, Luria-Bertani (LB) medium and LBKM medium (10 g/L tryptone, 5 g/L yeast extract, and 7 g/L KCl mediated by 100 mM 3-(N-morpholino) propanesulfonic acid (MOPS), adjusted to pH 7 using 5 M KOH) [32] were used for growth assays. Plasmids were maintained with 50 μg/ml spectinomycin, kanamycin, and chloramphenicol. A fresh 100-fold 6-APA solution was prepared by dissolving it in 1 M HCl, and appropriate concentrations of 6-APA and 1 mM IPTG were added to growth medium when applicable. All experiments were carried out at 37°C.

### Growth curve measurement

SDC, NPD, and cheater strains were inoculated into LBKM medium containing 50 μg/ml of antibiotics and 1 mM IPTG, incubated for 18 ~ 19 h, and then diluted 25-fold into fresh pre-warmed LBKM medium. After 2.5 h, they were diluted 5-fold and incubated for an additional 2.5 h, calibrating A600 to ~0.2. Cultures were mixed to create populations with ${R}_0$ = 0 to 1 (SDC and cheater strains) or NPD ratio from 0, 0.5, 1 (SDC and NPD strains). The populations were then diluted 40-fold into 200 μl of LBKM medium (with 1 mM IPTG) in a 100-well honeycomb plate, ensuring three replicates per condition for reproducibility. After 5 to 6 h of shaking to achieve an A600 of 0.15 ~ 0.2, an appropriate concentration of 6-APA was added to induce cooperation, with wells without 6-APA served as controls. Optical density measurements at a wavelength of 600 nm were performed using the Bioscreen C Automated Growth Curves Analysis System (FP-1100-C; Oy Growth Curves Ab) at 5 to 30 min intervals for 48 h.

### Characterization of the relative SDC ratio of the cells

At every given time point, a 10 μl or 20 μl aliquot of cell population from three replicate wells was diluted into 200 μl of pre-chilled cell counting buffer (0.9% NaCl with 0.12% formaldehyde, filtered using a 0.22 μm filter) and kept on ice-water bath until counting. To ensure accurate counting, the suspension was further diluted to achieve fewer than 5000 cells/second.

Bacterial cell counting was performed with a flow cytometer (Beckman; CytoFLEX S) with a flow rate of 60 μl/min and a run time of 60 s. The cell density of SDC (with GFP) and cheater was obtained using a 488-nm laser and a 561-nm laser, respectively (Fig. S10). Gains for the FSC, SSC, FITC, and ECD channels were set to 500, 500, 1500, and 1500, respectively. The final cell density, measured by either a plate reader or flow cytometer, was defined as the yield.

The SDC ratio was calculated by dividing the cell density of SDC by the total number of fluorescently labeled bacterial populations obtained from the flow cytometer. The proportion of susceptible SDC before adding antibiotics was defined as ${R}_0$, whereas the proportion of SDC at the final time point was denoted as ${R}^{\hbox{'}}$. The $\Delta R$ value was determined by subtracting ${R}_0$ from ${R}^{\hbox{'}}$.

### Sequential antibiotic treatment

To investigate the effects of repeated antibiotic exposure, the SDC strains were cultured in LBKM medium with 50 μg/ml of antibiotics and 1 mM IPTG. The cultures grew to an optical density (A600) of 0.15 ~ 0.2, after which they were exposed to 0.4 mg/ml 6-APA.

After the first round of 44 h antibiotic treatment, the cultures were combined and then diluted 200-fold into fresh, pre-warmed LBKM medium containing antibiotics and 1 mM IPTG. This process was repeated for three cycles. Optical density at 600 nm was monitored using the Bioscreen C Automated Growth Curves Analysis System (FP-1100-C; Oy Growth Curves Ab) at 15 min intervals over a 44 h period to represent cell densities.

### Growth and death rate calculations

The growth rate was determined during the exponential phase of the growth curve, defined as the maximum exponentially fitted rate across 120 consecutive minutes. In contrast, the death rate was calculated as the minimum exponentially fitted rate across 75 consecutive minutes following the induction of stress.

### Characterization of the growth curve of the 384-well plate when $\lambda =2$

To characterize the growth curve when $\lambda =2$, SDC and cheater strains were first inoculated into LBKM medium with antibiotics and 1 mM IPTG, incubated for 18 ~ 19 h. The overnight cultures were diluted 25-fold in fresh pre-warmed LBKM medium and incubated for 2.5 h to reach the exponential growth phase (A600 ~ 0.2). After further dilution (5-fold) and incubation for an additional 2.5 h, accurate cell counting was performed using a flow cytometer that had been flushed with 0.9% NaCl. Then the two bacterial strains were mixed in a 1:1 ratio directly on the flow cytometer.

The bacterial suspension was adjusted with 0.9% NaCl to achieve a $\lambda$ value of ~2, targeting around 1000 bacteria per 60 μl of suspension. This suspension was added to 25 ml of LBKM medium with antibiotics. Prior to dispensing into a 384-well plate, thorough mixing was achieved by aspirating and dispensing the solution 10 times with an electronic pipette (Eppendorf Xplorer plus, 12-channel), resulting in ~19.2 ml across all wells (50 μl per well). The plate was sealed with a breathable film (breath-easy, Diversified Biotech) to allow gas exchange. Optical density (A600) was measured every 10 min for 60 h using an Epoch 2 plate reader (Biotek) to monitor bacterial growth dynamics.

### Statistical analysis of bacterial lag phase and yield

To understand how dilution impacts bacterial growth, we employed statistical analysis of the obtained growth curve data when $\lambda =2$. For each well, we calculated the average A600 value between 300 and 1000 min, representing a period of stable growth. This average served as a baseline to identify the end of the lag phase. The lag phase was considered to conclude when the A600 value surpassed this baseline by a factor of one (indicating significant growth) and exceeded a minimum threshold of 0.05. This point signified the transition to exponential growth. The lag time, the time required for bacteria to adapt to the lower cell density caused by dilution and initiate active growth, was then calculated as the duration from the start of the culture to the end of the lag phase.

In addition to the lag phase analysis, bacterial yield was evaluated based on the A600 value at the final time point.

### Biofoundry SGSP procedures for self-destructive cooperation evolution

Experiments were conducted using a robotic workcell housed within the Shenzhen Infrastructure for Synthetic Biology (SISB, Fig. S5). This workcell contained a robotic arm on a 3.6-m track (Thermo Scientific, spinnaker mover), a liquid handling robot (Tecan, Freedom EVO 200) which was outfitted with a Roma robotic manipulation arm, a Liha 8-channel independent pipetter, a MCA96 with EVA adapter for 96-channel pipetting, a Te-Shake Silver shaker for high efficiency microplate oscillator. A microplate centrifuge (Agilent, G5582A), a microplate reader (Thermo Scientific, VarioskanLUX), an incubator (Thermo Scientific, Cytomat 2C 450 LIN Tos), a plate sealer (Kbiosystems, WASP), and a Xpeel seal pealer (Brooks Life Sciences, XP-A 230 V) were used. Software for control and data integration across these devices was provided by Momentum (Thermo Scientific, Version 6.0.2). Fluent control software (Tecan, Version 2.7) managed the liquid handling robot functions, including pipetting programs, temperature control, and labware transportation. The robotic platform facilitated the evolution of self-destructive cooperation in recombinant *E. coli* through a series of automated steps: segregation, growth, stress, and pool. Here was an example of a biofoundry experiment setup for three different antibiotic treatments.

Segregation.

1) Using flow cytometry, twice-activated overnight bacterial cultures (the activation method was consistent with that described previously) were mixed in predefined ratios and diluted to specific concentrations with culture medium within a 1.5 ml Ep tube.
2) The diluted culture and a reservoir containing 150 ml of LBKM medium supplemented with antibiotics and IPTG were then placed in a tube carrier and Te-Shake in the Freedom EVO 200 liquid handler robot, respectively.
3) A precise volume was transferred from the Ep tube to the reservoir to achieve the desired final concentration. Before each pipetting step, the Te-Shake mixed the solution in the reservoir for 10 s and used a 96-channel pipetting to aspirate and dispense the bacteria solution 3 times to create a well-mixed bacterial solution.
4) Finally, the robot transferred the strong diluted bacteria solution into six designated 384-well plates.
5) Following sealing of plates, the plates were transferred to an incubator set at 37°C and 1000 rpm for incubation (Movies S1).

Growth.

6) Following a 48 h incubation, a seal pealer removed the membranes from two out of the six 384-well plates (these two plates constituted one experimental set).
7) The Spark microplate reader then measured the yield (final cell density, A600) of the cultures, typically ranging from 0.4 to 0.8 (Fig. S6a). Wells with A600 readings exceeding 0.4 were selected for further analysis.
8) A custom program calculated the volume of bacterial culture needed to dilute them to a common target density (e.g. A600 = 0.04) based on their A600 values at the stationary phase.
9) The Freedom EVO 200 liquid handler robot in SISB performed this dilution process. The first row of a 96-well plate served as a blank control.
10) Following dilution, the WASP plate sealer sealed the two 96-well plates, which were then transferred to an incubator set at 37°C and 1000 rpm for further culturing using the robotic arm.
11) The remaining four 384-well plates were processed in sets of two, following the same experimental procedures described above (Movies S2).

Stress.

12) Bacterial cultures were incubated in 96-well plates at 37°C and 1000 rpm on Te-Shake Silver shakers for each set of two processed 384-well plates (refer to the previous section).
13) After reaching 75 min of incubation (Fig. S6d), the cultures underwent stress treatment with antibiotics. Each set received a specific concentration of antibiotics.
14) Following the addition of the stress, the plates were sealed with a plate sealer and returned to the incubator for a further 44 h of incubation at the same temperature and shaking speed (Movies S3).

Pool.

15) Following a 44 h incubation, the robotic arm retrieved each set of two processed 96-well plates (refer to the previous section).
16) A seal pealer removed the membranes from the plates.
17) 10 μl of bacterial culture in sets of two were collected from each well containing bacteria (excluding control wells) and pooled into a single Ep tube using the liquid handler (Movies S4).
18) This pooled culture was then analyzed using flow cytometry to determine cell number and ratio.

In parallel, the remaining bacterial solution was diluted back to the initial concentration used in the current experiment, preparing it for the next selection round.

Each round of the SGSP process, involving three different sets, took ~5 days. This setup allowed for up to three automated workflows to be completed in a single day, resulting in a total of nine different sets of experiments. By staggering the workflows across multiple days, a total of 18 workflows were conducted. This automated approach increased experimental efficiency 18-fold compared to manual methods, reducing the timeline from 4 months to just one week and eliminating human errors and inconsistencies.

### $\lambda$ calculation methods

Two distinct methods were employed to determine the $\lambda$, and their performance was compared against an established simulation. In the first method (Method 1), bacterial cells were counted using flow cytometry, and $\lambda$ calculated by averaging the number of bacteria per well based on the dilution factor. The second method involved counting empty wells in a 384-well plate and estimating $\lambda$ using the Poisson distribution formula, which related the frequency of empty wells to the average population size (Fig. S7a). Both methods were evaluated for accuracy and reliability, with results benchmarked against a simulation model to ensure robustness.

### ODE model for the synthetic SDC-cheater system

To capture the dynamic of the co-cultured strains, we modified the model first developed in Tanouchi 2012 MSB to characterize the growth curve of single strains [2]. Besides putting the equations of two strains together, we also added a nutrition equation to predict the maximal cell density that a system could reach, and also a resistance term of cell lysis to describe the resistant cells caused by phenotypic diversity. The full model could be written as follows:


$$ \frac{d{n}_S}{dt}=g{n}_S-{l}_S{n}_S $$

$$ \frac{d{n}_C}{dt}=g{n}_C-{l}_C{n}_{\mathrm{C}} $$

$$ \frac{dx}{dt}={\beta}_1\left(\frac{a}{\sigma_2+a}+\frac{x}{\sigma_3+x}\right)-\left({\gamma}_2+g\right)x $$

$$ \frac{db}{dt}={\beta}_2{l}_C{n}_C-{\gamma}_3b $$

$$ \frac{da}{dt}=-{\gamma}_5\frac{b}{\sigma_b+b}\frac{a}{\sigma_a+a} $$

$$ \frac{d{N}_{ut}}{dt}=-{\gamma}_4g\left({n}_S+{n}_C\right) $$

$$ g=g0\ \frac{\sigma_1}{\sigma_1+a}\cdotp \frac{\gamma_6{N}_{ut}}{\sigma_N+{N}_{ut}} $$

$$ {l}_S=\frac{\gamma_1\omega a}{\sigma_4+\omega a}+\frac{\gamma_7x}{\sigma_x+x} $$

$$ {l}_C=\frac{\gamma_1\omega a}{\sigma_4+\omega a} $$

Where ${n}_S\ {n}_C$ represented cell density of SDC and cheater; $x$ was the intracellular concentration of E protein; $b$ was the extracellular concentration of BlaM; $a$ was the extracellular concentration of 6-APA; ${N}_{ut}$ represented the nutrition left in the system; $g$ was the growth rate of both cooperator and cheater, as they were in a common growth condition, the growth rate was considered identical to both strain; ${l}_S\ {l}_C$ were the lysis rate of SDC and cheater. The parameters used in this research were either measured in related references or chosen in a biochemical reasonable range, and they were listed and described in Table S1. The initial condition was set to $n(0)=0.001,x(0)=0,b(0)=0$ antibiotic 6-APA was added to the system at 6 h by letting $a(0.4)=0\to 1$.

### Analyzing self-destructive cooperation evolution using the Price equation

The Price equation was expressed as: $\overline{W}\Delta \overline{R} = Cov\left(W,{R}_0\right)+\overline{W_i\Delta{R}_i}$. For self-destructive cooperation, we assumed that absolute fitness (*Wi*) was 0 for a homogeneous cheater population, whereas other ${R}_0$ received a fitness of 1. The ${R}^{\prime }$was 1 for a homogeneous group of SDCs and 0 for others. The $\Delta R$ decreased as ${R}_0$ varied, except when ${R}_0$ was 0 (Fig. S1a-d). The Poisson distribution led to stochastic fluctuations in group compositions, resulting in greater variances of ${R}_0$ and ${W}_i$. Using previously set parameters, we calculated $Cov\left(W,{R}_0\right)$ and $-\overline{W_i\Delta{R}_i}$. The point where these two values intersected was defined as ${\lambda}_C$.

## Results

### Theory predicted SDC maintenance in strong segregation

**Figure 1:**
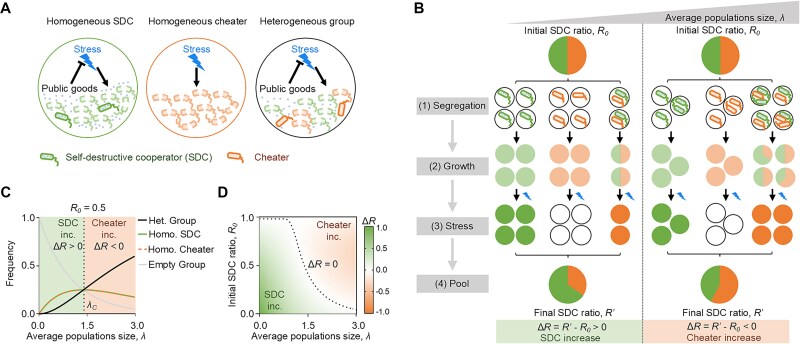
Theory predicted SDC maintenance under strong segregation. (**A**) SDC produced public goods (dots) that mitigated environmental stress (lightning bolts). Homogeneous SDC groups (Homo. SDC) benefited from mutual sacrifice, leading to high yield after stress. Homogeneous cheater groups (Homo. Cheater) failed to survive without SDCs, whereas heterogeneous groups allowed cheaters to exploit public goods, leading to cheaters proliferation. (**B**) A mixed population of SDCs and cheaters was segregated into homogeneous SDC, homogeneous cheater, and heterogeneous group under strong segregation. Smaller $\lambda$ increased the ${f}_{homo}^{SDC}$, boosting SDC survival and decreasing ${f}_{het}$ that favored cheaters. Strong segregation thus facilitated an increase in $\Delta R$ ($\Delta R\ge 0$). (**C**) For ${R}_0=0.5$, frequencies of homogeneous and heterogeneous groups were plotted against $\lambda$. With ${Y}_{homo}^{SDC}={Y}_{het}^{cheater}$, self-destructive cooperation maintenance required ${f}_{het}\le{f}_{homo}^{SDC}$ (dash line). (**D**) The $\Delta R$ after the SGSP process, calculated using Equation 2, was shown as a function of $\lambda$ and ${R}_0$. The dashed line represented the critical line $\Delta R=0$ separating SDC increase (SDC inc.) from cheater increase (Cheater inc.).

To illustrate the basic concept of how self-destructive cooperation was maintained in strong segregation, we considered the most extreme case of cooperation, where all the benefits were utilized by the non-sacrificing cheaters in heterogeneous groups composed of SDCs and cheaters. In this case, SDCs were assumed to entirely vanish (${Y}_{het}^{SDC}=0$) in these heterogeneous subgroups, whereas the cheaters maintained (${Y}_{het}^{cheater}$), regardless of the initial ratio between SDCs and cheaters. According to traditional group selection theory, the maintenance of cooperators required positively correlated yield and initial ratio of SDC in heterogeneous subgroups to balance the loss of SDC in every subgroup [11, 12, 33, 34]. However, SDCs were assumed to vanish in all heterogeneous groups, independent of their initial ratio. This independence implied that there was no inter-group advantage to offset the extreme intra-group cost of the SDC sacrifice, making it impossible to maintain SDCs according to traditional group selection theory (see Materials and Methods and Fig. S1a). In contrast, in homogeneous subgroups of SDCs, individual SDCs benefited from the sacrifice of other SDCs, leading to a high population yield $({Y}_{homo}^{SDC}$). Meanwhile, the homogeneous subgroup of cheaters vanished because there were no SDCs to protect the population under stress (${Y}_{homo}^{cheater}=0$) (Fig. 1A).

In our model, homogeneous subgroups contributed to the offspring of SDCs, whereas heterogeneous subgroups contributed to the offspring of cheaters. The maintenance of SDCs depended on the frequency of homogeneous SDC subgroups after segregation (${f}_{homo}^{SDC}$) as the frequency of heterogeneous subgroups (${f}_{het}$) was dependent on ${f}_{homo}^{SDC}$ (Fig. 1B). Given the initial frequency of SDCs before segregation (${R}_0$), we could compute the final ratio of SDCs after the SGSP procedures (${R}^{{\prime}}$) which could be expressed as:


(1) \begin{equation*} {R}^{\prime }=\frac{f_{homo}^{SDC}{Y}_{homo}^{SDC}}{f_{homo}^{SDC}{Y}_{homo}^{SDC}+{f}_{het}{Y}_{het}^{cheater}} \end{equation*}

The condition for self-destructive cooperation maintenance was a positive change in SDC ratio$(\Delta R\ge 0$), with $\Delta R={R}^{\prime }-{R}_0$. To eliminate the effect of frequency-dependent selection, we assumed that the yield of cheaters in heterogeneous groups was frequency-independent (${Y}_{het}^{cheater}={Y}_0$ where ${Y}_0$ was a constant). Additionally, under the assumption that SDC individuals in homogeneous groups produced the same amount of public goods as those in heterogeneous groups—benefiting both SDCs and cheaters equally within the same group—the yield of homogeneous SDC groups was equal to the yield of cheaters in heterogeneous groups (${Y}_{homo}^{SDC}={Y}_{het}^{cheater}={Y}_0$). Under these conditions, the criterion for SDC maintenance simplified to:


(2) \begin{equation*} \frac{f_{homo}^{SDC}}{f_{het}}\ge \frac{R_0}{1-{R}_0} \end{equation*}

In natural segregation cases, such as the division of biofilms, the formation of subgroups often followed the random sampling process, which led to a Poisson distribution of population sizes across the resulting subgroups [28, 29]. Consequently, the proportions of homogeneous and heterogeneous subgroups were solely determined by the average population size $\lambda$, defined by the average cell number across all subpopulations. In this scenario, strong segregation favored the formation of homogeneous SDC groups because smaller $\lambda$ (stronger segregation strength) led to larger ${f}_{homo}^{SDC}$ and smaller ${f}_{het}$ thus facilitating an increase in the $\Delta R$ ($\Delta R\ge 0$) (Fig. 1C). In the case of a balanced initial population (${R}_0=0.5$), the critical segregation strength (${\lambda}_C$) calculated using the equality in Equation 2, was approximately ${\lambda}_C\approx 1.5$ (dashed line in Fig. 1C). Moreover, lower ${R}_0$ necessitated a larger ${\lambda}_C$ (weaker segregation strength) for the self-destructive cooperation to persist (Fig. S2). This inverse relationship between the ${R}_0$ and the $\lambda$ on the$\Delta R$ was illustrated in a phase diagram (Fig. 1D).

Although our theoretical exploration showed that self-destructive cooperation could evolve in structured environments with strong segregation, experimental validation posed significant challenges. The primary challenge lay in creating a repeatable experimental framework that accurately modeled the synthetic SDC-cheater system. Additionally, population growth from one or two cells generated high variability in growth dynamics across a large number of subgroups. Therefore, a high-throughput pipeline with continuous monitoring and in-demand manipulation was required for the experiments [27]. In this paper, we overcame these challenges with synthetic bacterial strains and an automated biofoundry.

### Synthetic SDC-cheater system with engineered E. coli

**Figure 2:**
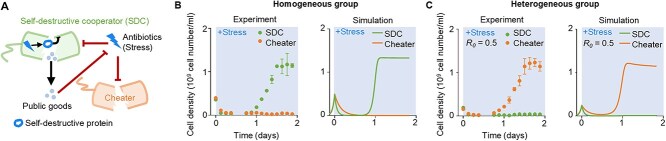
Engineered microbial SDC-cheater system. (**A**) The SDC strain underwent programmed cell death when exposed to antibiotic stress (blue lightning). This self-destruction released public goods (dots), such as the antibiotic-degrading enzyme BlaM, which mitigated the antibiotic stress. The cheater strain (red strain) underwent cell lysis without providing BlaM. (**B**–**C**) Experiments and simulations of the growth dynamics of engineered SDCs and cheaters in the homogeneous group (**B**) and heterogeneous group with an initial SDC ratio of 0.5 (**C**), with 0.4 mg/ml 6-APA added at time 0. The error bar represented the standard deviation of three biological replicates.

To probe the evolution of self-destructive cooperation, we employed a biological synthetic system with two engineered strains of *E. coli* (Fig. 2A, Fig. S3): one embodied the SDC phenotype and the other represented non-sacrificing cheaters. The SDC strain was engineered to undergo programmed cell death in the presence of antibiotics (6-APA). After cell death, the pre-expressed antibiotic-degrading enzyme (BlaM) was released into the environment, decreasing antibiotic concentration [2]. Meanwhile, the cheaters experienced cell lysis because of the antibiotic but did not provide any BlaM.

In a homogeneous group of SDCs, a minor subset inevitably survived environmental stress, facilitated by the public goods contributed by sacrificed SDCs, which served to mitigate the stress. Once the stress diminished to tolerable levels, the surviving SDCs regained their capacity to grow, ultimately giving rise to a new colony of SDCs with high yield (${Y}_{homo}^{SDC}$). However, in a homogeneous group of cheaters, the stress was not diminished effectively, resulting in a persistently inhibited cheater population at a reduced level yield (${Y}_{homo}^{cheater}$) (Fig. 2B). In heterogeneous groups, the imposition of external stress triggered the self-sacrifice of SDCs, which released public goods to mitigate stress. Initially, the non-sacrificing cheaters were suppressed by the stress. However, they recovered and began to grow earlier than the surviving SDCs, allowing the cheaters to dominate the population (${Y}_{het}^{SDC}\ll{Y}_{het}^{cheater}$) (Fig. 2C). The growth dynamics were then captured by a set of ordinary differential equations (ODE) (Table S1 and Materials and Methods). Furthermore, as long as the SDCs were able to generate sufficient public goods through self-sacrifice to lower the stress (${R}_0>0.3$) to a tolerable level, the yield of the cheaters became independent on ${R}_0$ but only dependent on the nutrients in the environment (Fig. S4). This phenomenon occurred because all SDCs were triggered to undergo self-sacrifice, leaving behind only negligible survivors that did not affect the yield of cheaters. In a typical antibiotic concentration of 0.4 mg/ml, we had ${Y}_{het}^{cheater}\approx{Y}_{homo}^{SDC}$ (Fig. 2), which allowed the application of Equation 2.

### Validation of SDC evolution using automated biofoundry

To continuously monitor growth dynamics and introduce the antibiotic stress at a consistent time setting for subgroups with high growth viability, we employed an automated biofoundry to control the procedures precisely (Fig. S5). This biofoundry utilized six devices, a robotic arm, two operating systems, and a flow cytometer, completing the SGSP process in 18 steps (see Materials and Methods).

**Figure 3:**
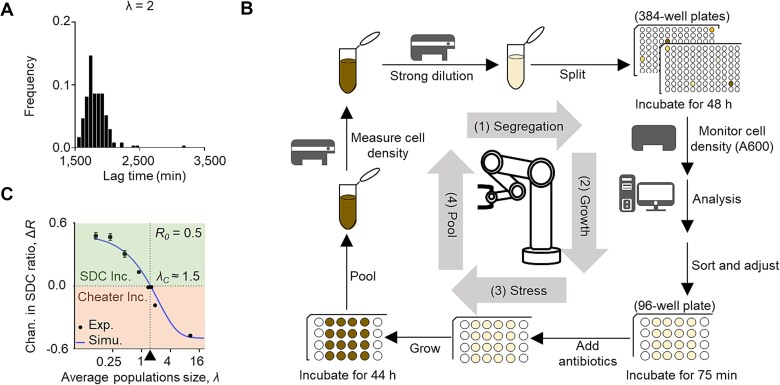
Biofoundry validation of SDC evolution under stronger segregation. (**A**) Frequency distribution of the lag times of the population dynamics for 3 ~ 4 colonies when $\lambda$ = 2. The lag time measurement was detailed in the Materials and Methods and Fig. S6a. (**B**) Illustration of an automated SGSP workflow with the biofoundry technology (see Materials and Methods for details). (**C**) Experimental and simulated results showed that the$\Delta R$ decreased with $\lambda$. A positive$\Delta R$ ($\Delta R$  $\ge$ 0) indicated an increase in SDCs, whereas a negative $\Delta R$($\Delta R$< 0) showed an increase in cheaters. The error bar represented the standard deviation for three technical replicates, and the antibiotic stress was set at 0.4 mg/ml 6-APA. Exp., experiment; Simu., simulation.

The ${R}_0\ \text{and}\ {R}^{\prime }$ were manually measured by flow cytometry. During the automated high-throughput SGSP experiments, individual subgroups were pre-cultured in multiple 384-well plates to the stationary phase, and the cell density was monitored through a plate reader (Fig. 3A and Fig. S6a-b). To ensure antibiotic stress was introduced uniformly at a similar cell density (0.1 < optical density (A600) < 0.3, Fig. S6c), each subgroup was diluted to an A600 of ~0.04. After 75 min re-culturing, a certain concentration of the antibiotic was then added to all the subgroups (Fig. S6d). The established automated workflow (Fig. 3B and Materials and Methods) offered an 18-fold improvement over the manual operation, reducing the time required for experiments from 4 months to just one week. This efficiency was achieved by running three different sets of experiments simultaneously, enabling up to 18 automated workflows within a week simultaneously eliminating human errors and inconsistencies (see Materials and Methods).

Using this automated protocol and synthetic SDC-cheater system, we investigated the$\Delta R$ at various segregation strengths (characterized by $\lambda$). The findings revealed a distinct pattern: when the initial group contained 50% SDC individuals (${R}_0=0.5$), the $\lambda$ threshold (${\lambda}_C$) was 1.5, indicating $\Delta R>0$ when $\lambda$ < 1.5 and *vice versa* (Fig. 3C and Fig. S7). This aligned well with theoretical predictions, suggesting that the evolution of SDC depended on the relative frequencies of homogeneous SDC and heterogeneous groups (Fig. S8a). Furthermore, we confirmed the ability of self-destructive cooperation to evolve and persist across multiple rounds of SGSP procedures (Fig. S8b). Collectively, these results supported the theory that self-destructive cooperation could maintain and evolve in a strong segregated environment.

To address concerns regarding the stability of the *E* gene, we conducted experiments to rule out the possibility of mutations or loss of the *E* gene under antibiotic treatment. The SDC strains were subjected to three rounds of 6-APA treatment, with recovery from cell death (Fig. S9a). If *E* gene mutations had occurred, the growth dynamics in later rounds would have shown slower death rates as a result of the survival of non-self-killing mutants, similar to what was observed in the growth dynamics of a mixture of SDC and a synthetic *E* gene mutation strain [2] (Fig. S9c-d). However, no significant decrease in death rate was observed across three rounds of antibiotic treatment with four replicates (Fig. S9b), confirming that the *E* gene did not mutate or become selected for the SGSP process.

### High stress facilitated SDC evolution

**Figure 4:**
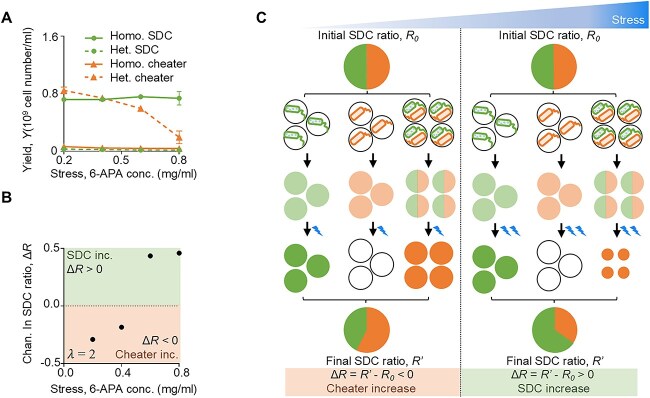
Stress facilitated SDC evolution in structured environments. (**A**) Experimental data of the yields at varying stress levels (6-APA concentrations). The yield of SDC in the homogeneous group (solid line with circle symbols) remained constant, whereas the yield of the cheater in the homogeneous group (solid line with triangular symbols) remained null. The yield of SDC in heterogeneous groups (dash line with circle symbols) remained null, and the yield of cheater decreased with stress intensity (dash line with triangular symbols). Homo. SDC, homogeneous SDC group; Het. SDC, heterogeneous SDC group; Homo. Cheater, homogeneous cheater group; Het. Cheater, heterogeneous cheater group. (**B)** The$\Delta R$ increased with stress intensity when $\lambda$ = 2.$\Delta R$  $\ge$ 0 signified an increase in SDCs, whereas$\Delta R$ < 0 denoted an increase in cheaters. The error bar in panels **A**-**B** represented the standard deviation of three biological replicates. (**C**) Illustration of how high stress facilitated SDC evolution. The reduced yield of heterogeneous cheater groups under high stress created selective pressure that favored the survival and proliferation of SDCs. Because SDCs produced public goods that alleviated stress, they were better equipped to survive and reproduce in stressful environments.

As the abovementioned theory, a simplified assumption was made: the yield of homogeneous SDC groups was equal to that of heterogeneous groups. However, this assumption did not hold when the stress level changed. We then investigated the impact of stress level (antibiotic concentration) on the yields of SDCs and cheaters. We found out that higher stress levels decreased cheaters’ yield within heterogeneous groups (Fig. 4A). This change was attributed to the limited capacity of public goods produced by SDCs to degrade more antibiotics in these mixed populations. Conversely, homogeneous SDC groups demonstrated a much higher ability to degrade antibiotics, rendering the yield of SDCs independent of the antibiotic concentration. This finding suggested that higher stress levels promoted the evolution of SDCs. The yield of SDCs in heterogeneous groups and homogeneous cheater groups was negligible across all antibiotic concentrations tested.

Further experiments employing the SGSP procedure with varying antibiotic concentrations revealed that even under weak segregation strength ($\lambda$  *=* 2), higher antibiotic concentrations were required to promote SDC maintenance in structured environments (Fig. 4B). This phenomenon can be explained by the observation that higher antibiotic concentrations reduced the yield of cheaters, thereby diminishing their fitness advantage (Fig. 4C). The comprehensive model, which incorporated yield data under varying antibiotic concentrations (Equation 1), allowed for theoretical recapitulation of the experimental findings. The effect of stress on SDC yields was directly implemented in the model without assuming any specific molecular mechanisms. We, therefore, concluded that high stress levels increased the advantage of homogeneous SDC subgroups by decreasing the fitness of cheaters in heterogeneous groups, thus creating a favorable environment for self-destructive cooperation to evolve.

## Discussion

This study shed light on the paradoxical emergence of self-destructive cooperation in structured environments. Although self-destructive cooperation was considered an altruistic trait [2, 35], its evolutionary path remained puzzling. However, the discovery of similar gasdermin pathways in bacteria and animal cells suggested an evolutionary connection [36], potentially supporting the “original sin” hypothesis of self-destructive cooperation origins, where death-related genes may have existed at the beginning of life [4]. Here, we proposed a mechanism where strong segregation and stressful environments promoted self-destructive cooperation evolution. Our theoretical deductions suggested that it occurred when the ratio of homogeneous SDC groups to heterogeneous groups fell below a critical value defined by initial SDC ratio (Equation 2 and Fig. 1D). These theoretical predictions were validated using a synthetic SDC-cheater system in *E. coli*. Mimicking self-destructive cooperation and strong segregation through an automated biofoundry, we successfully proved self-destructive cooperation evolution in stressful environments.

From a theoretical perspective, the prevalence of weak altruism was well-explained by group selection, which privatized the benefits of cooperation, thereby providing a fitness advantage over cheaters [17, 37, 38], as demonstrated by experiments conducted in structured environments [11, 18, 20, 21]. In contrast, the evolution of strong altruism, which imposed significant fitness costs on individuals, had traditionally been considered unlikely in structured environments, as strong altruists were thought to be inevitably outcompeted by selfish individuals [39]. In this study, we extended the theoretical framework of group selection by demonstrating that incorporating stochastic effects under conditions of strong segregation could specifically address the evolution of strong altruism. By focusing on self-destructive cooperation (SDC)—an extreme form of altruism where individuals completely sacrifice their fitness to produce public goods—we bridged a critical gap in evolutionary theory that previous models had overlooked. Our approach provided a more comprehensive understanding of how strong altruism could evolve under strong segregation, offering new insights into this long-standing theoretical challenge.

Our study significantly advanced the understanding of self-destructive cooperation (SDC) by building upon and extending foundational work in this field [2]. Although we adopted the SDC strain previously developed, we redesigned the cheater strain to exclude BlaM production genes, allowing for a more precise examination of cooperation and cheating dynamics. Unlike earlier studies, which primarily focused on the yield advantage of SDC strains, our work centered on the role of strong segregation in SDC evolution, revealing that subpopulations with fewer than three cells dominated under these conditions. To systematically investigate these dynamics, we developed a biofoundry workflow integrating high-throughput automated experiments with theoretical modeling, enabling us to rigorously test the effects of varying segregation strengths on SDC evolution.

Biofoundries represented a cutting-edge approach to laboratories, seamlessly integrating automation platforms for diverse workflows. Combining experimental approaches (mathematical modeling, computer-aided design, analytical software) with automated platforms enabled high-throughput workflows. This integration enhanced precision and stability through robotic arms and guide rails [40]. Our project utilized smaller cultivation volumes (microplates) to significantly increase throughput, addressing limitations of traditional methods and enabling rapid testing of numerous strains or variants. Although biofoundries have been applied in various fields, such as biosynthesis of plant-derived bioactive compounds [41] and directed protein evolution [42], their use in population genetics had been limited. This study pioneered the application of these technologies in population genetics, redesigning workflows for strong segregation conditions and demonstrating SDC evolution under such circumstances. The established automated workflow (Fig. 3B and Materials and Methods) offered an 18-fold improvement over manual operations, enabling faster processing and eliminating human errors. This suggested a significant advancement of automated technology, highlighting its efficiency and reliability.

Our findings highlighted the dependence of self-destructive cooperation evolution on various environmental factors. The $\lambda$ representing segregation strength, emerged as a critical factor in realistic scenarios with random sampling (Poisson distribution). Additionally, we observed a counterintuitive phenomenon: higher antibiotic concentrations, representing increased stress, facilitating SDC evolution. These insights were valuable for understanding the adaptive strategies employed by microorganisms in response to environmental stressors. Further exploration of SDC dynamics and its implications for microbial ecology held promise for advancing our understanding of cooperative evolution and designing intervention strategies to treat bacterial infections effectively.

In classical group selection theory, the Price equation was a pivotal analytical tool to evaluate the evolutionary potential of cooperative traits [10, 43, 44]. Under conventional segregation dynamics, wherein the population was uniformly allocated into subgroups with an equitably dispersed initial ratio of SDCs, the Price equation yielded a negative outcome, suggesting that SDCs could not be maintained through typical segregation mechanisms (Fig. S1a and Materials and Methods). Conversely, when strong segregation was imposed, homogeneous groups dominated the genotypes of subgroups, which resulted in a pronounced positive covariance between fitness (𝑊) and the initial SDC ratio ($Cov\left(W,{R}_0\right)$) and a low negative value of SDC fitness loss in each subgroup ($-\overline{W_i\Delta{R}_i}$). We recalculated the essential components of the Price equation as a function of the mean population size ($\lambda$), utilizing parameters established in Fig. S1b-d. As $\lambda$ increased (weaker segregation strength), $Cov\left(W,{R}_0\right)$ decreased and $-\overline{W_i\Delta{R}_i}$ increased. The critical value where the two terms intersected was found at ${\lambda}_C\approx 1.5$, which was consistent with our theoretical predictions and experimental observations (Fig. 1C and Fig. 3C). Thus, the persistence of SDCs under strong segregation did not violate the classic group selection theory, although this phenomenon had neither been demonstrated nor predicted before.

## Supplementary Material

ISMESI_clean_wraf043

Movies_S1_wraf043

Movies_S2_wraf043

Movies_S3_wraf043

Movies_S4_wraf043

Source_Data_wraf043

## Data Availability

All data generated or analyzed during this study are included in this published article and its supplementary information files, including the Source Data file.

## Preprint declaration

This manuscript was previously deposited as a preprint on *bioRxiv* under a CC-BY-NC-ND license, https://doi.org/10.1101/2024.10.15.618393, and was first posted on October 16, 2024.
