# Supplementary material for: Strong segregation promotes self-destructive cooperation
Source: ISME J. 2025 Mar 16;19(1):wraf043. doi: 10.1093/ismejo/wraf043 (PMC11948997; doi:10.1093/ismejo/wraf043)
Supplement: ISMESI_clean_wraf043 [file ismesi_clean_wraf043.pdf]

Supporting Information for

Strong segregation promotes self-destructive cooperation

Lingling Wen[1,2,5], Yang Bai[1,5], Yunquan Lan[3], Yaxin Shen[1,2], Xiaoyi She[1], Peng Dong[4], Teng Wang[1], Xiongfei Fu[1,2]*, Shuqiang Huang[1,2]*

*Corresponding author(s): Xiongfei Fu and Shuqiang Huang.
Email: xiongfei.fu@siat.ac.cn; shuqiang.huang@siat.ac.cn

This PDF file includes:

- Figures S1 to S10
- Tables S1
- Legends for Movies S1 to S4

Other supporting materials for this manuscript include the following:

- Movies S1 to S4

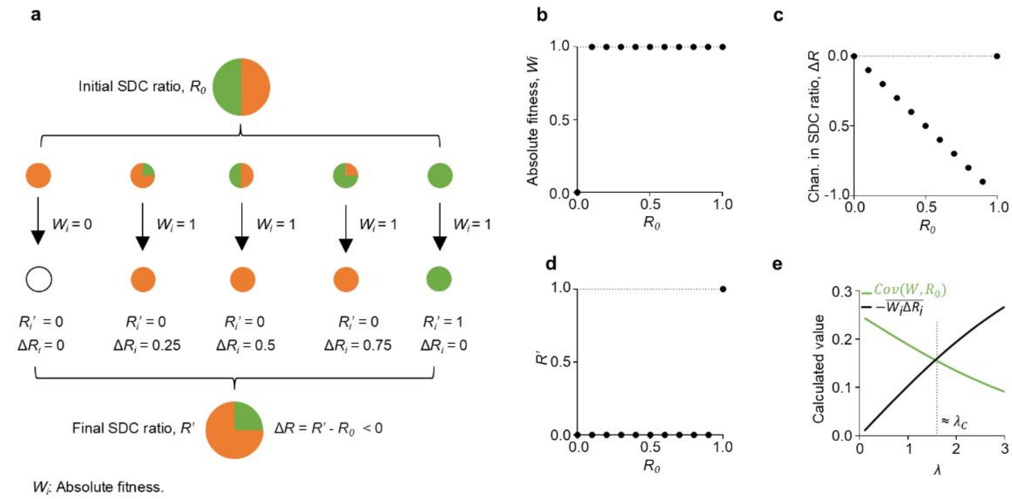

Fig. S1. Traditional group selection theory failed to predict the evolution of SDC in structured environments. a, We employed the Price equation to analyze self-destructive cooperation evolution. Initially, each individual (*i*) carried a genotype (R_i) designated as 1 for cooperators and 0 for cheaters (haploid). W_i varied based on the $\boldsymbol{R_0}$. The average genotype change ($\Delta\overline{R}$) was expressed as: $\overline{W}\Delta\overline{R} = \boldsymbol{Cov}(\boldsymbol{W}, \boldsymbol{R_0}) + \overline{W_i \Delta R_i}$. **b**, For SDC, W_i was 0 for a homogeneous cheater, whereas it was 1 for other $\boldsymbol{R_0}$ values. c, The ΔR exhibited a decreasing trend with increasing $\boldsymbol{R_0}$, except for $\boldsymbol{R_0}$=1. **d**, The *R'* was 1 for a homogeneous SDC and 0 for all other $\boldsymbol{R_0}$ values. Even under the most extreme scenario where covariance between R_0 and W_i equaled 1, the $\overline{W_i \Delta R_i}$ remained larger than covariance, hindering self-destructive cooperation evolution. **e**, To introduce significant variance, we proposed a mechanism involving extreme dilution of group populations (see Materials and Methods). We calculated $\boldsymbol{Cov}(\boldsymbol{W}, \boldsymbol{R_0})$ and $-\overline{W_i \Delta R_i}$ with decreasing $\boldsymbol{\lambda}$ (parameters set as described previously).

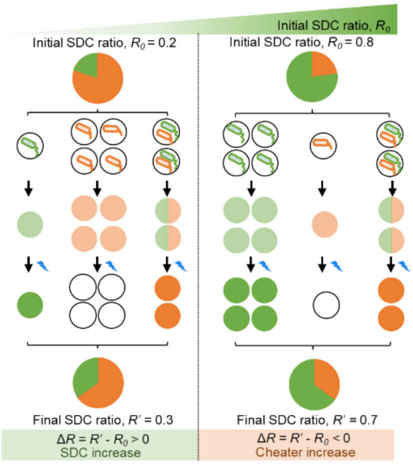

Fig. S2. Lower R_0 necessitated a larger λ_C for the SDCs to persist. In a fixed population structure, increasing the R_0 increased the frequency of homogeneous SDC groups, with the frequency of heterogeneous groups remaining unchanged. This implied that as R_0 increases, the proportion of homogeneous SDC groups relative to the total population also increased. However, when the groups were mixed after dispersion, this resulted in a lower R' compared to R_0 for higher R_0 values. Consequently, self-destructive cooperation could not evolve under these conditions.

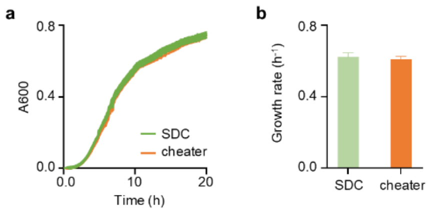

Fig. S3. Growth curves and growth rates of SDC and cheater strains without stress. a, Growth curves of SDC and cheater strains cultured separately without antibiotics. **b**, Calculation of the maximum growth rate for both strains (see Materials and Methods). Data was presented as mean ± sd from three biological replicates.

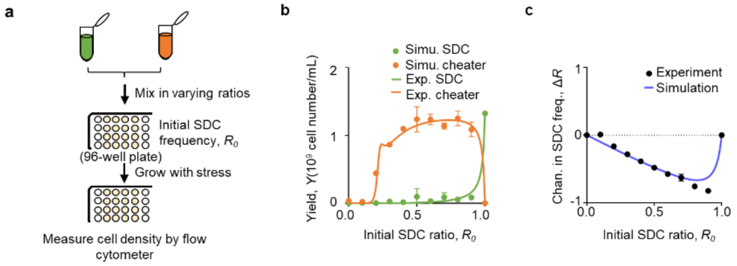

Fig. S4. Validation of constructed microbial self-destructive cooperation system. a, Schematic representation of the experimental setup for growing a mixture of SDC and cheater strains in varying ratios under stress conditions without segregation. **b**, Yields of SDC and cheater in different group compositions. Experimental results (points, n = 3) were compared with simulation of the ODE model (lines, Materials and Methods) in the presence of 0.4 mg/mL 6-APA. **c**, The relationship between $\boldsymbol{R_0}$ and $\boldsymbol{\Delta R}$ after stress induction. Error bar represented the mean ± s.d. for three biological replicates.

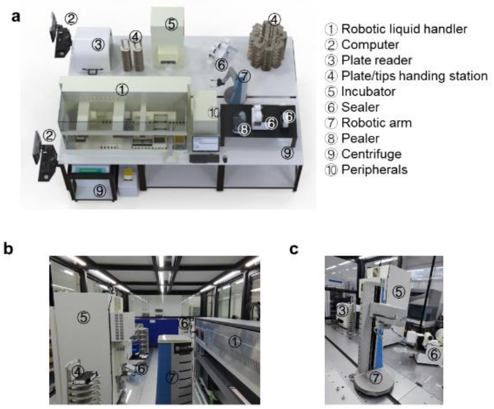

Fig. S5. Shenzhen Infrastructure for Synthetic Biology (SISB) biofoundry layout for SDC evolution. Illustrated the design and configuration of a biofoundry within the SISB specifically adapted for studying self-destructive cooperation evolution. **a**, Schematic representation of the automated biofoundry. It depicted various core and peripheral instruments interconnected by a central robotic arm. Scheduling software managed the entire system, coordinating the operation of individual components. **b-c**, Physical drawings of the automation platform from two different perspectives.

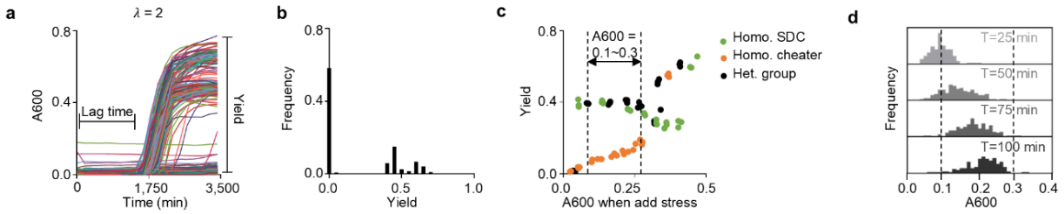

Fig. S6. Necessity of an automated biofoundry for SGSP procedures. a, Variation in growth dynamics following dilution. Growth curves of multiple subpopulations when $\boldsymbol{\lambda = 2}$ at 3,500 min (about 60 h) of culturing in a 384-well plate. Lag time was defined as the time to reach exponential growth (as shown in Fig. 3a), and yield was defined as the final cell density (A600). **b**, Frequency distribution histogram of the yield data when $\boldsymbol{\lambda = 2}$, with intervals of 0.05 A600 units. **c**, The impact of adding 0.4 mg/mL 6-APA at different A600 on yield. Two conditions were compared: monoculture (including homogeneous SDC and homogeneous cheater) and co-culture (heterogeneous group, $\boldsymbol{R_0 = 0.5}$). The dashed line indicated that when stress was applied within this A600 range, the yields remained relatively stable. Homo. SDC, homogeneous SDC group; Homo. cheater, homogeneous cheater group; Het. group, heterogeneous group. **d**, After the growth step, bacteria were re-cultured in a shaker, and their cell density was measured using a microplate reader within the automated biofoundry every 25 min. The dashed line indicated that the A600 was within the target range for adding the antibiotic.

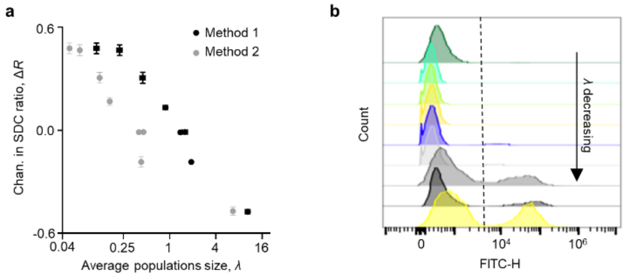

Fig. S7. λ calculation methods and final SDC ratio distribution. a, Two methods were used to determine the $\boldsymbol{\lambda}$ when $\boldsymbol{R_0 = 0.5}$ (see Materials and Methods). Method 1, indicated by the black dots, was believed to be more accurate due to the potential for underestimation in Method 2 (grey dots). This underestimation was likely caused by strong dilution impacts during the experiment, which may have hindered the recovery of some bacteria. Data represented the mean ± s.d. for three technical replicates. **b**, Distribution of $\boldsymbol{R'}$ across varying $\boldsymbol{\lambda}$ values using flow cytometer.

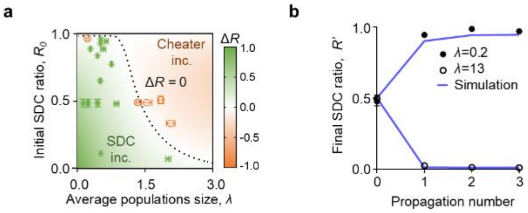

Fig. S8. Confirmation of SDC evolution in small population sizes. a, Validation of the $\boldsymbol{\Delta R}$ after the SGSP process, calculated according to equation 1, as a function of $\boldsymbol{\lambda}$ and $\boldsymbol{R_0}$ (as shown Fig. 1d). **b**, Comparison of experimental results (points, n = 3) with simulation results (lines) calculated using equation 2, across multiple cultivation rounds in either strong segregation ($\boldsymbol{\lambda = 0.2}$) or weak segregation ($\boldsymbol{\lambda = 13}$), in the presence of 0.4 mg/mL 6-APA with $\boldsymbol{R_0 = 0.5}$. Error bar represented the mean ± s.d. for three technical replicates.

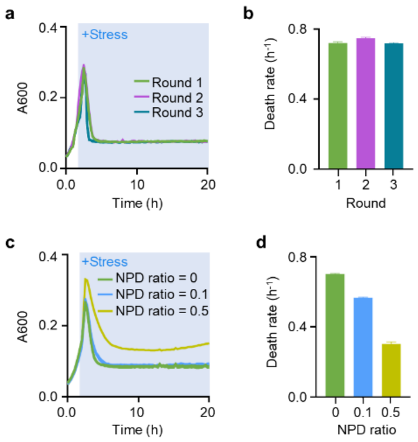

Fig. S9. Growth dynamics of SDC strains under sequential antibiotic treatment and mixed population of SDC and NPD strains. a, Growth dynamics of the SDC group across three sequential rounds of antibiotic treatment, showing consistent growth patterns. **b**, Maximal death rates in the SDC group, which remain unchanged across rounds. **c**, Growth dynamics of mixtures of SDC and NPD strains at different initial NPD ratios (0, 0.1, and 0.5) under antibiotic treatments. **d**, Varying maximal death rates observed in the mixed populations of SDC and NPD strains.

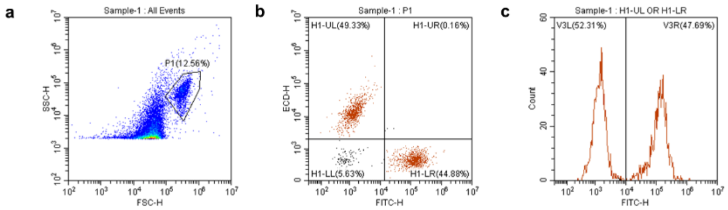

Fig. S10. Gating strategy for flow cytometry analysis. a, Particles within region P1 were identified as bacterial cells based on forward scatter height (FSC-H) and side scatter height (SSC-H) characteristics. The established *E. coli* self-destructive cooperation system was labeled with a constitutively expressed green fluorescent protein (GFP) and red fluorescent protein (mCherry), respectively. This allowed quantification of their ratio through flow cytometry analysis. An example flow cytometry plot with a mixed population was shown. **b**, The plot of FITC-H versus ECD-H further differentiated the populations based on their respective fluorescent signals. Cells within H1-UL and H1-LR sub-populations were designated as cheater and SDC strains, respectively. H1-UL represented the GFP-negative, mCherry-positive population (cheaters), whereas H1-LR represented the GFP-positive, mCherry-negative population (SDCs) based on their fluorescence profiles. **c**, By comparing the number of SDC cells to the total number of fluorescently labeled bacterial populations, the $\boldsymbol{R_0}$ was obtained.

Table S1.

ODE Model parameters

Parameter	Symbol	Value	Reference
Growth rate	g0	0.90 /h	Calculated from this study
Half-maximal constant for a degradation of 6-APA by beta-lactamase	σ_b	0.28 molecules/mL	This study
Half-maximal constant for a degradation by 6-APA	σ_a	0.10 mM	This study
E protein synthesis rate	β_1	18 molecules/cell	Copy number of pColE1 strain
BlaM production rate	β_2	10 molecules/cell	Copy number of p15A strain
Maximum cell lysis rate of cheater	γ_1	0.48 /h	Calculated from this study
Degradation rate of E protein	γ_2	200 molecules/h	This study
Intrinsic degradation rate of BlaM	γ_3	0.09 molecules/h	This study
Yield nutrition consumption	γ_4	0.80 OD/mM	Calculated from this study
Max degradation rate of 6-APA by BlaM	γ_5	0.06 mM/h	This study
Max nutrition - dependent growth rate	γ_6	1.50 mM/h	Calculated from this study
Maximum cell lysis rate of SDC	γ_7	2.60 /h	Calculated from this study
Half-maximal constant for growth inhibition of 6-APA	σ_1	0.02 mg/mL	This study
Half-maximal constant for E protein synthesis by 6-APA	σ_2	32.60	Relative BlaM expression of P_{ampC} in an ampD$^+$ strain (SN0301) to SN03 was 65.20（*30*）.
Half-maximal constant for E protein positive feedback	σ_3	2	This study
Half-maximal constant for 6-APA-dependent lysis	σ_4	2	This study
Half-maximal constant for nutrition-dependent growth	σ_N	0.60 mM	Max nutrition was 1.20 mM
Half-maximal constant for BlaM -dependent lysis	σ_x	0.004 molecules/cell	This study
Effective killing efficiency of antibiotic	ω	10	This study

Movies S1 (separate file). Automated segregation in SGSP procedures for SDC evolution experiments. This video outlined the automated process for steps 2 through 5, including segregation, mixing, and incubation of bacterial cultures. The experimental setup was designed to study SDC evolution in a microbial SDC-cheater system.

Movies S2 (separate file). Automated growth in SGSP process for SDC evolution experiments. This video demonstrated experimental procedures, detailing the workflow used to measure bacterial growth, perform dilutions, and continue culturing under controlled conditions as part of the SGSP process.

Movies S3 (separate file). Automated stress in SGSP procedures for SDC evolution experiments. This video demonstrated experimental procedures, outlining the workflow for adding stress to bacterial cultures as part of the SGSP process.

Movies S4 (separate file). Automated pooling in SGSP workflow for SDC evolution experiments. This video demonstrated the experimental procedures for steps 15 through 17, outlining the workflow for pooling bacterial cultures as part of the SGSP process.
